# The Validity of the Holtzman Inkblot Technique: New Indices

**DOI:** 10.3389/fpsyg.2021.621669

**Published:** 2021-09-22

**Authors:** James Dawe, Raymond C. Hawkins II, Marco Lauriola, Falk Leichsenring, Lina Pezzuti

**Affiliations:** ^1^Department of Dynamic and Clinical Psychology, Faculty of Medicine and Psychology, Sapienza University of Rome, Rome, Italy; ^2^School of Psychology, Fielding Graduate University, Santa Barbara, CA, United States; ^3^Department of Social and Developmental Psychology, Sapienza University of Rome, Rome, Lazio, Italy; ^4^Department of Psychosomatics and Psychotherapy, University of Giessen, Giessen, Germany

**Keywords:** holtzman inkblot technique, rorschach (comprehensive system), correlations, personality assessment, validation

## Abstract

**Objective:** The present study examines the validity of 11 new Holtzman Inkblot Technique indices. These were chosen from Exner’s Comprehensive System (RCS) indices using two criteria: first, they had to be valid according to meta-analysis, and second, they must be computed using the HIT standard scoring system.

**Methods:** Both techniques were administrated with a retest interval from 1 to 7days to a sample of 139 subjects (63 males and 76 females) from the general population. The validity of the new indices was studied through Pearson correlation (*r*) with the corresponding RCS indices.

**Results:** Nine of the 11 new indices (R-HIT, F%-HIT, M-HIT, m-HIT, C'-HIT, Blends-HIT, PureH-HIT, DQ+HIT, and X-%-HIT) showed significant correlations with Rorschach scales, confirming our hypotheses. The correlation ranged from a minimum of 0.144 to a maximum of 0.414.

**Conclusions:** The results provide support for the validity of the new HIT indices and have important implications for both clinical and research fields.

## Introduction

During the development of the Holtzman Inkblot Technique (HIT), Holtzman et al. (1961) created 22 new scoring variables from the Rorschach’s literature. The rationale was that psychologists who were more familiar with the Rorschach could obtain most of its base variables from the HIT. For example, the Rorschach determinant Chromatic Color is traditionally divided into three categories according to the balance between two characteristics of the blots, form and chromatic color, in determining the percept: Form-Color (FC), where the form features of the blot primary and the color is of second importance; Color-Form (*CF*), where color is the main determinant and form is of secondary importance; and pure Color response (C), where no form is involved (Exner, 2003). What about the HIT? First of all, there is no distinction between chromatic and achromatic color. Second, in the HIT, the three distinct categories are transformed into three scores (FC=1, *CF*=2, and C=3) and then summed to create the total score for the variable Color (C). Last, there is only one total score in the HIT, while in the Rorschach, there are three different total scores (FC, *CF*, and C) based on how many times FC, *CF*, and C were scored in the protocol. How do these two systems influence interpretation? In the Rorschach Comprehensive System (RCS; Exner, 2003), the three categories have different meanings and converge in several different indices (e.g., Complexity Ratio, Blends, representing the proportion of answers, where two or more determinants were scored respect all other answers). Only the total C score is interpreted in the HIT, with a loss of information regarding the single score. The same applies to all 22 HIT variables: only total scores are interpreted, with consequences for both clinical and research applications.

The HIT is a performance-based technique for the assessment of personality. Like the Rorschach, the HIT uses inkblots, some of which are chromatic and others achromatic, and is individually administrated in the traditional position with the examiner sitting next to the subject. However, the two techniques show some differences. First of all, the HIT uses two parallel forms of 45 inkblot each (plus two examples in common in the two forms), compared with 10 inkblots in the Rorschach. The guidelines call for only one response per blot, followed by a brief standardized inquiry each time, opening to the possibility of a group administration of the test. Finaly, unlike the Rorschach, HIT’s inkblots show different degrees of asymmetry.

The HIT was commended on its excellent reliability for a performance-based technique (Holtzman et al., 1961; Gamble, 1972; Holtzman, 1975; Anastasi, 1982; Lilienfeld et al., 2000; Holtzman and Swartz, 2003; Darolia, 2016), its ability to differentiate individuals with psychopathology from healthy controls and among different psychopathologies (Holtzman et al., 1961; Moseley, 1963; Megargee and Velez-Diaz, 1971; Leichsenring, 1990, 1991; Darolia, 2016), its sensitivity to detect the developmental changes across the lifespan (Thorpe and Swartz, 1965, 1966; Swartz et al., 1967; Darolia, 2016), its maintaining a constant number of total responses to the protocols (Lilienfeld et al., 2000), and for the possibility of group administration (Darolia, 2016; Holtzman, 1988; Panek et al., 1983). All these features represent advantages over the Rorschach in research and clinical assessment, especially when it comes to a situation, where many subjects have to be tested (e.g., academic, job, and military selection). Moreover, HIT administration is easy to learn and does not rely on the interpretive skill of the administrator, so beginners can learn how to use it without a long training (unlike many tests such as the Thematic Apperception Test or the Rorschach). However, two main issues have been highlighted: the validity of the HIT is still an open controversy (for a comprehensive review, see Darolia, 2016), and the interpretative system shows some limitations (Zubin, 1972; Dana, 1973) due to the way HIT variables are scored and, in our opinion, the absence of more complex indices. In the present study, we will focus on the latter, hoping that this discussion will also enhance the validity of the test.

In literature, researchers have tried to overcome the limitations of the interpretative system either using an adapted version of Rorschach coding variables to HIT protocols or developing entirely new variables from HIT scores. However, no complex indices (variables resulting from the combination of multiple scores) were developed, as in the Comprehensive System (e.g., Blends), derived from the combination of multiple HIT variables. Consistent with the first approach, Doris et al. (1963) took the Rorschach’s Form Quality variable, which assesses the degree of correspondence between the form of the concept and the form of the corresponding inkblot, and rated it using a 4-point scale, from very good (4) to very poor (1). The same study found that second and third-grade children belonging to different groups based on their anxiety levels (measured with the Test Anxiety Scale for Children) differed on the Form Level rating. The same trends were also found for Human Movement, Location, and Space, all coded following corresponding procedures used in the Rorschach. Another attempt was carried out by Endicott and Jortner (1966), who aimed to develop a reliable and valid measure of depression. They administered the HIT, along with other measures of depression, to two groups of subjects, 90 hospitalized patients and 40 outpatients, as a control group. All HIT protocols were scored using the traditional Rorschach system for color, and a Depression Rating Scale, which measured depression on a 5-point scale from minimal to extreme depression, was used to test the validity of the scoring method. The results showed a statistically significant correlation with *CF* (*r*=−0.23) in the hospitalized group, confirming previous findings with the Rorschach, but not in the outpatient group. The study suggested that the correlation between HIT color and depression was at best weak. Similarly, Cooper and Caston (1970) studied the relationship between fantasy, represented by the M response, and physical activity (5-min period on a exercycle), in order to clarify the direction of the relationship. The authors used a 15 card version of the HIT and scored the protocol of 29 adults (all men) using three different versions of the variable Movement: the traditional Rorschach M response, the HIT Movement (M) variable (which include human, animal, and inanimate activities), and a new version of this HIT variable where only human activity was rated according to Holtzman’s 5-point score scale for Movement (0, 1, 2, 3, and 4). The experiment consisted in the administration of the first 15 inkblots of Form A before the physical activity and the first 15 inkblots of Form B during the physical activity. Not only did the intercorrelation matrix show highly significant positive correlations between corresponding Forma A and B variables but also all variables showed an increase in their scores during the physical activity in comparison with before the exercise. Several authors in past decades have applied Rorschach scoring variables to HIT protocols with good results (Megargee and Cook, 1967; Bowers and van der Meulen, 1970; Bowers, 1971; Lefcourt et al., 1972; Domino, 1980; Lockwood et al., 1981; Rosegrant, 1982; Prokop, 1983; O’Neill et al., 1984).

Although less extensively used, another approach to overcome the limitations of the HIT interpretative system has been improving the original scoring system to develop new variables. For example, Sanders et al. (1968) transformed the three scores of Color (1, 2, and 3) into three different variables as shown in Table 1. They aimed to investigate the developmental trend of the Color total score and the three new variables in a longitudinal study of 323 children. Their results showed a consistent decline for all scores except for one of the new variables, FC. Leichsenring (1990, 1991, 1999) applied the same procedure to the HIT variable Pathognomonic Verbalization (V), which is based on a 4-point scale according to the degree of psychopathology, and created five new variables, one for each score level (DV1, DV2, DV3, and DV4), plus a fifth, which is the sum of level 3 and 4 (DV34). He demonstrated the clinical validity of this new system and showed the diagnostic utility of using the single score of the HIT instead of only the total score of V.

**Table 1:** Example of scoring of the variable Color (C) and the new variables C1, C2, and C3 (according to Sanders et al., 1968).

Inkblot	Color (C)	FC (1)	*CF* (2)	C (3)
1	0	0	0	0
2	1	1	0	0
3	3	0	0	1
4	2	0	1	0
…	…	…	…	…
45	1	1	0	0

In the present study, we aimed to address the limitations of the HIT interpretation system, integrating the first approach (i.e., application of Rorschach categories) and the second strategy (i.e., use of HIT scores to develop new variables by using the original HIT scoring system) to create and study the validity of new complex indices derived from the RCS. In this way, we may improve the HIT’s utility in both clinical and research fields. In doing so, we administrated the standard version of the HIT and the Rorschach according to Exner’s Comprehensive System (Exner, 2003). We selected the new indices, choosing among Exner’s indices using two criteria: first, they had to be valid according to the Mihura et al. (2013) meta-analysis, and second, they had to be applicable to the HIT standard scoring system. In Table 2, we describe the seven HIT variables that we used, while in Table 3, we report the selected Rorschach indices, how they were calculated, and how they can be obtained using the HIT scoring system. Holtzman et al. (1961) correlated HIT variables with the Rorschach scores derived by converting Beck’s measures to their equivalent HIT variables. For example, Localization (L) in the HIT system is scored on a 3-point scale: a score of 0 indicates that the subject used the whole blot, a score of 1 is given when a large part of the blot is used, and a score of 2 when small details are used. So, Holtzman et al. transformed Beck’s W to a score of 0 like the variable Localization (L) in the HIT. Similarly, D was converted to a score of 1 and Dd and d to a score of 2. The scores were then summed across all responses and then correlated with HIT variable L.

**Table 2:** Description of seven traditional HIT variables.

HIT core variables	Score	Descriptions
Rejection (R)	0–1	Number of inkblots without a scorable response
Location (L)	0–1–2	Proportion of inkblot used to form the percept
Form Definiteness (FD)	0–1–2–3–4	How is definite the form of the concept expressed in the answer
Form Appropriateness (FA)	0–1–2	Goodness of the fit of the form of concepts to the form of the inkblot
Color (C)	0–1–2–3	Represent the importance of color in determine the percept
Shading (Sh)	0–1–2	It is scored when the subject uses the shade in the inkblot for the creation of the answer
Movement (M)	0–1–2–3–4	Amount of energy ascribed in the response
Integration (I)	0–1	Presence of a relationship between two or more separate part of the blot organize in a larger whole
Human Content (H)	0–1–2	Presence of human elements or whole humane figure
Animal Content (A)	0–1–2	Presence of animal elements or whole animal figure

*The definitions above are drawn from Holtzman’s manual (Holtzman et al., 1961)*.

**Table 3:** Creation of the new HIT indices.

Rorschach Indices	Description	New HIT Indices	Description
Number of Response (R)	Total number of the response given by the subject	R-HIT	45 minus number of rejections
Form% (F%)	The proportion of F response respect all answers	L-HIT	The proportion of responses, where Color (C), Shading (Sh), and Movement (M), gain 0, and Form Definiteness (FD) gain at least 1 point respect all answers
Human Movement (M)	Total of percepts with human activity	M-HIT	Total of percepts scored at least 1 on both Movement (M) and Human Content (H)
Inanimate Movement (m)	Total of percepts with the images of nonliving objects in motion	m-HIT	Total of percepts with a score of 0 on both Human (H) and Animal Content (A) and at least one point in Movement (M)
Achromatic Color (C')	Total answers in which pure achromatic color feature of the inkblot was used	C'-HIT	Total number of answers with a score of 3 in the black and white inkblots
Sum of Shading (SumShd)	Sum of Diffuse, Vista, Texure, and Achromatic Color	SumShd-HIT	Total number of answers with a score on FD of 0 and on Sh of 2, plus the score of C'-HIT
Complexity Ratio (Blends)	Total number of percepts scored with more than one determinant/R	Blends-HIT	Total number of answers with a score on at least 2 among C, Sh, and M, divided the number of responses (R-HIT)
Whole Realistic Human (PureH)	Total number of percepts with total realistic human	PureH-HIT	Total of answers with a score of 2 on Human Content
Synthesize Response (DQ+)	Total number of percepts were two or more objects are in relation to each other	DQ+HIT	Total number of percepts, which meet two criteria: FD>1 and I=1
Form Quality Score: Appropriate (WDA%)	Total number of answers with an Ordinary-elaborated (FQ+), ordinary (FQo), or unusual (FQu) Form Quality (FQ) in the locations W and D, divided the number of responses with W or D locations	WDA%-HIT	Total number of responses with a score of at least 1 in FA and a score of 0 or 1 in L, divided the number of answers with a core of o or 1 in L
Form Quality Score: Distorted (X-%)	The proportion of FQ- respect all other answers	X-%-HIT	Total of answers with a score of 0 on FA divited the number of responses (R-HIT)

The conversion procedure used in the current study is opposite to the one performed by Holtzman et al. (1961), except for Human Movement (M-HIT), where we used the same equation. We disaggregated HIT scores in order to compute the selected RCS variables. This procedure produces two important consequences. In fact, according to Rushton et al. (1983), aggregated scores tend to be more reliable and valid (e.g., aggregated scales are more reliable than single items). So, the new HIT indices should show smaller correlation coefficients than if we had converted RCS scores into their more dimensional HIT counterparts, and also smaller correlations than those observed by Holtzman et al. (1961). As in Holtzman et al. (1961) study, and following the suggestion of Kinder (1992), we decided to transform some of the RCS scores in order to control for the effect of the Number of Responses (R), by dividing the RCS index for R and multiplying by 100. The new RCS indices are distinguished from the traditional ones by the symbol %. We decided to keep both versions of the RCS indices for a comparition. This procedure was computed for: Human Movement (M), Inanimate Movement (m), Achromatic Color (C'), Sum of Shading (SumShd), Whole Realistic Human (PureH), and Synthesized Response (DQ+). Moreover, following Meyer et al. (2001) we decided to compute Form% instead of Lambda (%), due to the fact that this index is more suitable to use in parametric analysis.

We expected that the new indices would show a positive correlation with the corresponding Rorschach indices. Moreover, we hypothesized that:

1. R-HIT will show a positive correlation with the RCS index Number of Responses (R).
2. F%-HIT will show a positive correlation with the RCS index Form% and a negative correlation with Blends.
3. M-HIT will show a positive correlation with the RCS indices Human Movement (M%) and Blends.
4. m-HIT will show a positive correlation with the RCS index Inanimate Movement (m%).
5. C'-HIT will show a positive correlation with the RCS index Achromatic Color (C'%).
6. SumShd-HIT will show a positive correlation with the RCS index Sum of Shading (SumShd%).
7. Blends-HIT will show a positive correlation with the RCS indices Blends and M, and a negative correlation with L.
8. PureH-HIT will show a positive correlation with the RCS index Whole Realistic Human (PureH%).
9. DQ+HIT will show a positive correlation with the RCS index Synthesized Response (DQ+%).
10. WDA%-HIT will show a positive correlation with the RCS index Form Quality Scores: Appropriate.
11. X-%-HIT will show a positive correlation with the RCS index Form Quality Scores: Distorted.

## Materials and Methods

### Participants

The HIT and the Rorschach were administered to a sample of 139 Italian participants (63 males and 76 females). The participants’ ages ranged from 18 to 60years (*M*=38years, *SD*=12.12years). Educational levels ranged from primary school (5years of education) to a university degree (18years) with a mean of 13.51years (*SD*=3.7). Part of the sample was composed of university students recruited from undregraduate psychology classes of five psychologists who collected the data and who had been thoroughly trained in the administration of both tests. All participants joined this research voluntarily. The Ethical Committee of the Department of Dynamic and Clinical Psychology, University of Rome “Sapienza” approved all aspects of this study.

### Instruments

The 45 inkblots of the HIT (Form A) plus two practice blots were individually administered and then coded by the first author following Holtzman’s manual guidelines (Holtzman et al., 1961). The participants were allowed to provide only one answer for each card, and a brief and standardized inquiry followed every response. All protocols were hand-coded according to seven HIT variables following Holtzman’s manual guidelines: Rejection (R), Form Definiteness (FD), Color (C), Shading (Sh), Movement (M), Integration (I), and Human Content (H). Unlike the Rorschach, the HIT scoring system is quantitative with different weights for every variable. The single scores of each variable are then summed across the 45 cards. The first author transformed those variables into the new indices.

The Rorschach was administered and coded following Exner’s Comprehensive System guidelines (Exner, 2003). It consists of 10 symmetrical inkblots created by Herman Rorschach in 1921: five black and white, two black and white with red elements, and three completely colorful. The administration is divided into two phases: first, collecting the responses to all 10 inkblots, followed by a second phase inquiry about those responses to gather information on the location, where the participant had seen the percept, what it was, and which features of the blot had suggested it. All protocols were coded using an online program available on the Virtual Psychology site: Rorschach Assistance Program (ver. 3). For the present study, only 11 indices were considered: Number of Responses (R), Lambda (L), Human Movement (M), Inanimate Movement (m), Achromatic Color (C'), Sum of Shadings (SumShd), Complexity Ratio (Blends), Whole Realistic Human (Pure H), Synthesized Response (DQ+), and Form Quality Scores: Appropriate (WDA%) and Distorted (X-%).

The five psychologists were in contact and were in charge of finding the subjects, following two criteria: Each of them had to collect the same number of protocols for males and females (to avoid any bias due to a sample with a gender imbalance); and, all subjects had to be in the age range 18–60years. Participants were not paid for their participation. The HIT and the Rorschach were administered in separate sessions. The interval between sessions ranged from a minimum of 1day to a maximum of 7days. The order of test administration was counterbalanced such that approximately half of the participants took the Rorschach (or the HIT) at the first session. A number of protocols were coded by the first author and the data collector. After 90% agreement was reached on the totals of each scored variable, subsequent protocols were coded by the data collector only.

### Data Analysis

Pearson correlations were calculated between HIT and Rorschach variables, and the null hypotheses were tested with one-tailed significance tests. Pearson’s correlations were interpreted according to Cohen’s (1988) guidelines. A correlation coefficient of 0.10 is thought to represent a small effect size; a coefficient of 0.30 is considered as moderate; a coefficient equal to or greater than 0.50 is thought to represent a large effect size.

Inter-rater reliability for the six HIT variables used to compute the new indices were computed on 80 protocols randomly drawn from the collected protocols. All coefficients were interpreted following Nunnally and Bernstein's (1994) indications.

## Results

In Table 4 are reported the Means (M) and Standard Deviations (SD) of all variables considered in the study.

**Table 4:** Descriptive statistics of HIT new indices and rorschach comprehensive system (RCS) indices.

	HIT			RCS	
	*M*	*SD*		*M*	*SD*
R-HIT	43.38	2.8	R	24.57	6.42
F%-HIT	0.37	0.16	F%	0.51	0.17
M-HIT	8.36	5.3	M	2.15	1.68
m-HIT	1.59	1.52	M%	8.95	6.96
C'-HIT	0.36	0.69	m	0.59	0.95
SumSh + C'-HIT	0.97	1.20	m%	2.33	3.62
Blends-HIT	0.14	0.11	C'	1.17	1.50
PureH-HIT	8.75	4.35	C'%	4.57	5.80
DQ+HIT	8.49	5.69	SumSh+C'	1.29	1.65
WDA%	0.75	0.28	SumSh+C'%	4.99	6.21
X-%HIT	0.34	0.29	Blends	0.09	0.087
			Pure H	2.4	1.96
			PureH%	9.86	7.46
			DQ+	4.7	3.21
			DQ+%	19.31	12.46
			WDA%	0.76	0.11
			X-%	0.25	0.11

The nine correlation coefficients for inter-scorer reliability are reported in Table 5. They range from 0.756 to 0.987. These results are similar to those obtained by Holtzman et al. (1961). For a comparison, the Holtzman et al. (1961) correlations are also included in this tables.

**Table 5:** Inter-rater reliability of the six HIT variables.

	Present study	Holtzman et al. (1961)
L	0.967	0.993
FD	0.982	0.995
FA	0.756	0.98
C	0.981	0.96
Sh	0.967	0.97
M	0.933	0.98
I	0.986	0.89
H	0.907	0.995
A	0.987	0.995

Table 6 reports all correlations between the HIT new indices and, as an external criterion, Rorschach’s indices, along with their significance levels. Nine new HIT variables (i.e., F%-HIT, M-HIT, m-HIT, C'-HIT, Blends-HIT, PureH-HIT, DQ+HIT, and X-%-HIT) showed significant correlations with the corresponding RCS indices ranging from low to moderate. These findings suggest a partial overlap that could be indicative of convergent validity.

**Table 6:** Correlations of HIT new indices with Rorschach comprehensive system indices with and without correction for number of responses (R).

	Number of responses (R)			
R-HIT	0.202^**^			
	F%	Blends		
F%-HIT	0.286^***^	−0.383^***^		
	M	M%	Blends	
M-HIT	0.252^**^	0.206^**^	0.379^***^	
	m	m%		
m-HIT	0.116	0.144^*^		
	C'	C'%		
C'-HIT	0.145^*^	0.153^*^		
	SumSh+C'	SumSh+C'%		
SumSh + C'-HIT	0.098	−0.071		
	Blends	F%	M	M%
Blends-HIT	0.414^***^	−0.221^**^	0.090	0.020
	Pure H	PureH%		
PureH-HIT	0.311^***^	0.221^**^		
	DQ+	DQ+%		
DQ + HIT	0.282^***^	0.274^**^		
	WDA%			
WDA%-HIT	−0.111			
	X-%			
X-%-HIT	0.199^**^			

*RCS indices transformed shows a % next to them*.

* *p<0.05;*

** *p<0.01;*

*** *p<0.001*.

R-HIT showed a positive correlation (*r*=0.202) with Number of Responses (R), suggesting that higher scores on this new HIT index correspond with a greater number of responses in a Rorschach protocol, which represent the ability to respond inkblots with many ideas.

F%-HIT showed a positive correlation (*r*=0.286) with Form% (F%), suggesting that higher scores on this new HIT index correspond to increases in the value of L, which measures the tendency to avoid or to focus on complexity, subtlety, or nuance. Moreover, there was a significant negative correlation between F%-HIT and the Complexity Ratio (Blends; *r*=−0.387), which suggests that an increase in the score on this new HIT index corresponds to a decrease in this CS variable that measure psychological complexity.

M-HIT showed a positive correlation with Human Movement (M; *r*=0.252), M% (*r*=0.206), and Complexity Ratio (Blends; *r*=0.379). So, higher scores on this new HIT variable correspond to an increase in these Rorschach indices, which is indicative of mental abilities, such as planning, imagination, and empathy (M), and psychological complexity (Blends).

The new HIT index m-HIT showed a positive correlation with m% (*r*=0.144), suggesting that higher scores on this new HIT index correspond to an increase in this Rorschach variable (m%) that measures mental distraction or agitation, often as a reaction to a moderate to severe stressor.

C'-HIT showed a positive correlation with both Achromatic Color (C'; *r*=0.145) and C'% (*r*=0.153). This relationship suggests that an increase in the responses based solely on achromatic color as the determinant corresponds with an increase in this RCS index that measures the presence of negative emotions.

Blends-HIT showed a positive correlation with Complexity Ratio (Blends; *r*=0.414) and a negative correlation with Form% (F%; *r*=−0.221). So, the increase of scores on this new HIT variable corresponds to an increase in the Rorschach index, which is indicative of psychological complexity (Blends) and a decrease in the Rorschach index, which is representative of the tendency to avoid or to focus on complexity, subtlety, or nuance (F%).

PureH-HIT showed a positive correlation (*r*=0.311) with the Whole Realistic Human (Pure H) index and PureH% (*r*=0.221). So, increasing scores on this HIT new variable corresponds to an increase in the number of Pure H responses. High scores on this Rorschach index suggest that the subject’s perceptions of others are reality-based.

DQ+HIT showed a positive correlation with Synthesized Response (DQ+; *r*=0.282), suggesting that higher scores on this new HIT index correspond with an increase in DQ+ in the Rorschach, meaning where two or more objects are in a meaningful relation, which is indicative of the quality of processing.

X-%-HIT showed a positive correlation with Form Quality Score: Distorted (X-%; *r*=0.199). So, higher scores on this new HIT index correspond to an increase in the Rorschach index, which is indicative of distorted perception.

## Discussion

This study aimed to analyze the validity of 10 completely new HIT indices created using the original HIT scoring system, through correlation with the corresponding Rorschach scales as external criteria. For this purpose, both tests were individually administered with 1–7days retest interval to a sample of 139 subjects. This study showed several significant correlations between the HIT new variables and the Rorschach indices, confirming 9 of the 11 hypotheses, suggesting the presence of a relationship and supporting the validity of the HIT new variables. The correlations with the corresponding Rorschach indices were statistically significant for seven new HIT indices.

As hypothesized, R-HIT showed a low but significant correlation with the corresponding Rorschach index Number of Responses (R). This relationship may suggest that R-HIT could be interpreted as a measure of the tendency to respond with many ideas to an ambiguous stimulus. This result confirms previous Holtzman et al. (1961) findings, where Rejection (R), the opposite of R-HIT, showed a negative correlation with Number of Responses (R).

F%-HIT showed a low but significant correlation with the corresponding Rorschach index Form% (F%). This relationship may indicate that this new HIT index could be interpreted as a measure of the tendency of the subject to avoid complexity (high scores) or deal with it (low scores) through focusing on details or nuances. This interpretation was supported by the negative correlation with the Complexity Ratio (Blends), which showed a reverse relationship with CS’s F%. Indeed, F% increases with the number of answers based solely on form and decreases with percepts based on other determinants such as color or movement, features that raise the scores of Blends. Moreover, on the interpretative level, Blends and F% are opposites (Exner, 2003): the former increase with high levels of psychological complexity and the latter increase with the avoidance of complexity.

In the RCS, Human Movement (M) is one of the most studied variables, and there is a considerable agreement on its interpretation (Exner, 2003; Porcelli and Kleiger, 2015). Since M is present in multiple clusters, it assumes different meanings (i.e., it is indicative of a form of sophisticated and well organized superior mental activity, empathy, creativity, and mentalization). As in the Holtzman et al. (1961) study, our hypothesis regarding M-HIT was confirmed, and it seems to share these interpretations through its low but positive significant correlation with Human Movement (M) and M%, and with another Rorschach index: Blends. These relationships support the validity of this new HIT index as a measure of mental abilities. Moreover, if we go deeper into the single components of Blends we find that, together with color and shading, M represents the determinants that occur to raise Blends which could clarify the positive correlation between M-HIT with this index.

Both m-HIT and C'-HIT showed a low positive correlation with the corresponding RCS indices. The small effect size of this relationship may be explained by the way the two new indices were computed, which could lead to an underestimation of their scores. In the first case, HIT inanimate movement was the sum of responses with a score of 0 in Human Content (H) and Animal Content (A) and at least a score of 1 in Movement (M), and this sum excluded all responses, where inanimate movement occurred with human or animal content (“A human staring at a flying airplane”). Regarding C'-HIT, this was the sum of responses to the white and black inkblots with a score for Color (C); however, there were also achromatic colors in most of the other inkblot stimuli. So, all responses that use achromatic color in these other inkblots were not detected by C'-HIT scores. To further support this interpretation, these categories should be directly coded on the protocols and not computed from HIT scores.

Noteworthy is the moderate positive correlation between Blends-HIT and Blends. This relationship could imply that this new HIT variable may be interpreted as a measure of psychological complexity. Moreover, Blends-HIT showed a negative correlation with F%, which may suggest that the psychological complexity measured by Blends-HIT may be related to the tendency of the subject to avoid the complexity (high scores) or deal with it (low scores) through focusing on details or nuances. This result is in line with the negative correlation between F%-HIT and Blends.

The results confirm our hypothesis regarding PureH-HIT, showing a positive correlation between PureH-HIT and the Whole Realistic Human (Pure H) and H%. This may suggest that this new HIT index may be interpreted as a measure of the ability to have a whole, reality-based perception of the other. This finding is not surprising. Fernald and Linden (1966) previously found that a person with strong social interest tends to give more human content responses. In their study, the variable was coded using the standard Rorschach method, where human details, human-like figures, and whole humans receive only one point. Moreover, Holtzman et al. (1961) obtained a correlation of 0.62 between HIT human content and Beck’s converted Human Content.

DQ+HIT, as expected, showed a low but significant correlation with CS’s DQ+ and DQ+%. This correlation may support the validity of this new HIT index, and it suggests that DQ+HIT can indicate the quality of processing.

As hypothesized X-%-HIT showed a positive correlation with Form Quality Score: Distorted (X-%). This relationship may suggest that this new HIT index may be interpreted as a measure of distorted perception. However, since there is not previous literature for a comparition with our study’s results and given the low effect of the association, these positive correlations should be considered with caution.

## Conclusion

Ever since the development of the HIT, the focus has been mainly on the test’s psychometric properties. Our main goal in this study has been to provide an alternative to the Rorschach, keeping all the good qualities of the HIT while strengthening its psychometric basis (Holtzman et al., 1961). However, how HIT variables are coded leads to a loss of information. Previous research highlighted the utility of the HIT as a research tool and coped with this limitation by either applying the Rorschach coding system to HIT protocols or creating new variables using a HIT coding system (Endicott and Jortner, 1966; Bowers and van der Meulen, 1970; Cooper and Caston, 1970; Bowers, 1971; Lefcourt et al., 1972; Domino, 1980; Lockwood et al., 1981; Rosegrant, 1982; Prokop, 1983; O’Neill et al., 1984). In line with this approach, we used the HIT variables and their scores to develop more sophisticated indices, taking inspiration from Exner’s Comprehensive System. Our findings support the validity of nine new variables through their significant low to moderate correlations with the corresponding Rorschach indices. However, we would expect a higher correlation since the two tests are similar. Before attributing the low correlations to a lack of validity of the HIT, we should be trying to code both tests with the same coding system and by using a new brief version of the HIT, where inkblots are selected using the information from the literature and based on empirical data. An example of a brief version was proposed by Hawkins et al. (2019). The HIT Brief Revisited Form is composed of three parallel forms with six inkblots each.

In the present study the attempt to control for the effect of the Number of Responses (R) on other Rorschach scores seems to have had an impact on the correlation between m-HIT and m and between PureH-HIT and PureH. Of the different ways through which the Number of Responses (R) can be controlled in the RCS, we decided to transform the indices in percentage of R, as Holtzman et al. (1961) did. However, following the suggestion of Exner (1992), we decided to perform analyses with both versions of these RCS indices, since transformation may lead to a distortion in the findings, and this may also be the case in our study. For the future research, best results may be obtained by controlling R during the test administration, as this would drastically reduce any distortion of the results due to transformation. Pianowski et al. (2021) demonstrated through a comparison of scores obtained using both RCS and the new Rorschach Performance Assessment System (R-PAS), that an administration procedure to reduce R variability had little impact on scores.

This study had some limitations. The first is that the data were collected from a convenience sample of participants recruited from the general population. Consequently, there could be a volunteer bias in that those with a higher interest in psychological inquiries would have participated in the study, especially the students. Plus, the participants were the acquaintances of the five different psychologists, so the study findings may not be generalizable. A second limitation is that interrater reliability coefficients were not calculated on all protocols for both HIT and Rorschach for the five different psychologists collecting the protocols and coding them, although a single psychologist had trained them with repeated joint training sessions. A third limitation may be represented by the correlation with another performance-based test, whose validity has also been questioned, which should lead the reader to interpret the results with caution, even if the correlations are with valid scores according to the Mihura et al. (2013) meta-analysis. More studies are needed to sustain our findings and the validity of the new indices using self-reports or direct observational behavior to have additional evidence of validity. Even if the present study results cannot be seen as a proof of validity, they give an insight into the possible meaning of the new indices that could be useful for the design of future studies. A third limitation concerns the use of less aggregated scores than used by Holtzman et al. (1961). As we said before, less aggregated scores (e.g., two vocabulary items from an IQ test) tend to show lower correlation coefficients in comparison to correlations using more aggregated counterpart scores (e.g., two Verbal IQ scales; Rushton et al., 1983). This is likely why our observed correlations were lower than Holtzman et al. (1961). A last limitation is that some of this new HIT indices could be under- or over-estimated. For example, M-HIT was the sum of responses, where a score of at least 1 in the HIT variables Movement (M) and Human Content (H) was given. However, since we worked with the summary score data and not directly with the protocols, there is not certainty that the movement was related to the human content. In the answer “A man with a dog running next to him” the movement is associated with the Animal Content (A) and not the Human Content (H), but we still would give a score of 1 in M-HIT because a score of 2 was given to Human Content (H) and a score of 3 to Movement (M). The same limitation could be applied to the new HIT indices m-HIT, C'-HIT, SumShd-HIT, and PureH-HIT. In order to obtain a more accurate measure of these indices the HIT scoring system would have to be modified.

Despite these limitations, the present study opens a new perspective for HIT research and the future of this test. The development of new and more informative indices leads to important consequences for both clinical applications, where the utility of these HIT new indices for assessment can be determined and research in personality, psychopathology, and treatment. Moreover, some of the results could be generalized to the R-PAS version of RCS variables such as F%, M, m, C', SumShd (YTVC’ in the R-PAS), Blends%, X-% (FQ-% in R-PAS) and PureH, which are the same in the two methods. Concerning the remaining variables: DQ+ is similar to the new variable Sy, though the latter also includes DQv/+; and, WDA% represent the inverse of the variable WD-% in the R-PAS, so they are conceptually the same. So, these variables should not show significant differences across the two methods. Thus, this study may be considered as a first step toward developing a robust interpretive system for the HIT based not only on the traditional 22 variables but also through adding more complex new indices. More research is needed to support our initial findings and clarify the meaning of the new indices. A potential approach for future studies could involve a new brief version of the test with two answers for each inkblot and the inclusion of new indices, keeping the original idea of Holtzman et al. (1961) of a coding system based on scores, but modifying the approach with which the variables are coded or by introducing new codes. This strategy can strengthen the validity of the HIT. The second line of research could involve using a clinical sample to test the HIT’s utility within a diagnostic assessment. Leichsenring (1990, 1991, 1999) has already opened this approach with his research on new variables derived from the HIT variable Pathognomonic Verbalization (V), and it would be interesting to replicate this research with the new indices.

## Data Availability Statement

The raw data supporting the conclusions of this article will be made available by the authors, without undue reservation.

## Ethics Statement

The studies involving human participants were reviewed and approved by the Comitato Etico di Dipartimento, Department of Dynamic and Clinical Psychology. The patients/participants provided their written informed consent to participate in this study.

## Author Contributions

JD developed the research design, conceived the original idea, carried out the analysis, and wrote the manuscript with the support from LP, RH, FL, and ML. LP supervised the project and contributed to the interpretation of the results. ML supervised the analysis. RH and FL provided critical feedback and contributed to the final form of the manuscript. All authors contributed to the article and approved the submitted version.

## Conflict of Interest

The authors declare that the research was conducted in the absence of any commercial or financial relationships that could be construed as a potential conflict of interest.

## Publisher’s Note

All claims expressed in this article are solely those of the authors and do not necessarily represent those of their affiliated organizations, or those of the publisher, the editors and the reviewers. Any product that may be evaluated in this article, or claim that may be made by its manufacturer, is not guaranteed or endorsed by the publisher.
